# Single and cumulative exposure to psychosocial work conditions and mental health among young adults

**DOI:** 10.1093/eurpub/ckad015

**Published:** 2023-02-15

**Authors:** Samira de Groot, Karin Veldman, Benjamin C Amick, Ute Bültmann

**Affiliations:** Department of Health Sciences, Community and Occupational Medicine, University of Groningen, University Medical Center Groningen, Groningen, The Netherlands; Department of Health Sciences, Community and Occupational Medicine, University of Groningen, University Medical Center Groningen, Groningen, The Netherlands; Department of Epidemiology, Fay W Boozman College of Public Health, University of Arkansas for Medical Sciences, Little Rock, AR, USA; Winthrop Rockefeller Cancer Institute, University of Arkansas for Medical Sciences, Little Rock, AR, USA; Department of Health Sciences, Community and Occupational Medicine, University of Groningen, University Medical Center Groningen, Groningen, The Netherlands

## Abstract

**Background:**

The consequences of a single point-in-time compared to cumulative exposure to psychosocial work conditions (PWCs) for young adults’ mental health have received relatively little attention. This study investigates (i) the associations between single and cumulative exposure to adverse PWCs at ages 22 and 26 with mental health problems (MHPs) among young adults at age 29 and (ii) the effect of early life MHPs on MHPs at age 29.

**Methods:**

Data were used from 362 participants in the TRacking Adolescents’ Individual Lives Survey (TRAILS), a Dutch prospective cohort study with 18-year follow-up. PWCs were assessed at ages 22 and 26 with the Copenhagen Psychosocial Questionnaire. Internalizing (i.e. depressive and somatic complaints, anxiety) and externalizing MHPs (i.e. aggressive and rule-breaking behaviour) were measured by the Youth/Adult Self-Report at ages 11, 13, 16, 19, 22 and 29. Regression analyses were conducted to examine the associations between single and cumulative exposure to PWCs and MHPs.

**Results:**

Single exposure to high work demands at ages 22 or 26 and high-strain jobs at age 22 were associated with internalizing problems at age 29; the association attenuated after adjustment for early life internalizing problems but remained significant. No associations were found between cumulative exposures and internalizing problems. No associations were found between single or cumulative exposures to PWCs and externalizing problems at age 29.

**Conclusions:**

In view of the mental health burden in working populations our findings call for early implementation of programmes targeting both work demands and MHPs to keep young adults working.

## Introduction

Mental health problems are a major risk factor for lower work functioning, sickness absence and work disability and come with high costs for the individual, employers and society.^1–5^ To date, we do know that adverse psychosocial work conditions (PWCs), such as high levels of job demands and low levels of job control, negatively affect the mental health of working young adults.^6–10^ Yet, the consequences of single point-in-time and cumulative exposure to PWCs for young adults’ mental health have received relatively little attention. Young workers consistently report adverse PWCs more frequently than their older colleagues.^7^^,^^11^^,^^12^ A better understanding of the impact of single point-in-time and cumulative exposure to PWCs on young adults’ mental health is of great importance for policy and practice to support informed decisions for implementing programmes to keep young adults working.

Only a few studies have examined the impact of cumulative exposure to PWCs on adult mental health. Although the studies differ in study population composition and study design,^13–16^ they all indicate that cumulative exposure over time to adverse PWCs impacts mental health more than a single point in time. Stansfeld *et al*.^13^ found in an older working population that multiple episodes of job strain over 5 years doubled the risk of major depressive disorder. In a young Swiss working population (ages 21–31) that consisted of student workers in vocational training, a positive association was found between cumulative trajectories of adverse PWCs and somatic complaints.^14^ These two studies in vastly different working age populations suggest workers who experience cumulative exposure have an increased risk of major depressive disorder^13^ and reported more somatic complaints^14^ than those with a single exposure. To date, however, to the best of our knowledge, no studies have examined the impact of cumulative exposure to PWCs on internalizing problems (i.e. depressive and somatic complaints, anxiety) among a young general working population. With regard to the study by Stansfeld *et al*. on major depressive disorder among the older workforce, it is questionable whether results from two decades ago are replicable to young workers anno 2020. Earlier research, has shown that externalizing problems (i.e. aggressive and rule-breaking behaviour) among young workers were associated with work outcomes.^2^^,^^17^ Yet, to the best of our knowledge, no study has examined the impact of single or cumulative PWCs on externalizing problems. Therefore, disentangling the effect of single and cumulative exposures to adverse PWCs on today’s young workers’ internalizing and externalizing problems contributes to an understudied area.

Furthermore, most studies on PWCs and mental health problems have neglected to consider workers’ early life mental health status, albeit several studies have controlled for baseline mental health. The few studies that did address early life mental health problems indicated that mental health problems during childhood, adolescence and/or young adulthood have long-term consequences for a workers’ career and mental health.^2^^,^^18^ Also, we do know that childhood internalizing and externalizing problems are rather stable over time and may extend into adulthood.^2^^,^^19^ Based on these studies, we expect that early life mental health problems might attenuate the observed associations between PWCs and subsequent internalizing and externalizing problems.

The present study aims to (i) examine the associations between single and cumulative exposures to adverse PWCs and subsequent internalizing and externalizing problems among young adults and (ii) investigate if adjusting for early life internalizing and externalizing problems attenuates the associations of single and cumulative exposures to adverse PWCs and subsequent internalizing and externalizing problems. We will apply a life course approach to better understand the impact of adverse PWCs on young workers’ internalizing and externalizing problems. The most recent 2019/20 data from the 18-year follow-up TRacking Adolescents’ Individual Lives Survey (TRAILS) will be used.

## Methods

### Study design and sample

We used data from seven waves of TRAILS, an ongoing prospective population-based cohort study on the psychological, social and physical development of adolescents and young adults. The participants were selected in March 2001 when five municipalities from the three Northern provinces in the Netherlands invited all children born between 1 October 1989 and 30 September 1991^20^^,^^21^ to participate. The children and one or both parents provided informed consent and the Dutch Central Committee on Research Involving Human Subjects approved all study protocols. The baseline study took place in 2001 including 2230 children (76.0% from the initial sample) with a mean age of 11.1 years (SD = 0.55). Second wave children were 13.5 years (SD = 0.53, *N* = 2149, response rate 96.4%), third wave adolescents were 16.3 years (SD = 0.69, *N* = 1816, 81.4%), fourth wave adolescents were 19.1 years (SD = 0.58, *N* = 1881, 84.3%), fifth wave adults were 22.3 years (SD = 0.65, *N* = 1775, 79.6%), sixth wave adults were 25.7 years (SD = 0.60, *N* = 1618, 72.6%) and seventh wave adults were 28.9 years (SD = 0.60, *N* = 1230, 55.2%). We included TRAILS participants who had data on their mental health status at age 29, who had a paid job, worked more than 12 h per week at ages 22 and 26 and who provided information about their PWCs at ages 22 and 26. The cut-off of 12 h work per week refers to the definition of Statistics Netherlands concerning the active labour force.^22^ The final study sample consisted of 362 participants out of 1230 (29.4%). Of the excluded participants, 78.2% did not had a paid job at ages 22 and 26 or worked <12 h per week, 12.9% had missing data on mental health at age 29, and 8.9% who worked more than 12 h per week had missing data on PWCs.

### Measures

*Mental health problems* were assessed at age 29 with the Dutch version of the Adult Self-Report (ASR).^23^ The ASR is a valid and reliable measurement tool.^23^ The ASR is one of the most widely used assessment instruments for adult emotional and behavioural problems.^24^ Participants indicated their emotional and behavioural problems over the last 6 months on a three-point Likert-scale ranging from 0 = not true, 1 = somewhat or sometimes true, 2 = very true or often true. Two scales ‘internalising problems’ and ‘externalising problems’ are derived from the ASR.^24^ Internalizing problems comprises anxious/depressed, withdrawn/depressed and somatic complaints. Externalizing problems comprises aggressive and rule-breaking behaviour. Standardized scores were created following Achenbach with higher scores indicating higher levels of internalizing or externalizing problems.^23^ To improve interpretability, the standardized scores were multiplied by 50 to set the scale range from 0 to 100. Participants with missing data were listwise deleted.^23^

*Psychosocial work conditions* were measured at ages 22 and 26 with the short version of the Copenhagen Psychosocial Questionnaire (COPSOQ II).^25^ The COPSOQ is a research-based survey that has been used at workplaces and in research.^26^ The COPSOQ is recognized as a valid and reliable risk assessment instrument by the International Labour Organization and the World Health Organization.^26–30^ Four 2-item subdomains of PWCs were included: quantitative demands, work pace, decision authority and possibilities for development. The items were scored on five response categories ranging from 0 to 4. The subdomain scores were calculated by summing up the item scores with higher scores indicating higher levels of quantitative demands, work pace, decision authority and possibilities for development.

Following Karasek’s Job Demand-Control model,^31^ we built ‘work demands’ and ‘decision latitude’ measures. Work demands (Cronbach’s alpha coefficients 0.52 and 0.67 for age 22 and 26, respectively) were operationalized by the sum of two subscales: quantitative demands and work pace. Decision latitude (Cronbach’s alpha coefficient 0.66 for ages 22 and 26) was operationalized by the sum of two subscales: decision authority and possibilities for development. If more than 20% of the items were missing, the total scale score was set to missing. The total scale scores were then grouped into tertiles: low, medium and high. The two dimensions of work demands and decision latitude were combined to create the Job Demand-Control quadrant. Work demands and decision latitude were first split at their median and subsequently cross-classified^32^ creating: high-strain (high work demands, low decision latitude), low-strain (low work demands, high decision latitude), passive (low work demands, low decision latitude) and active jobs (high work demands, high decision latitude). Cumulative exposure to PWCs was created by adding the work demands at age 22 to the work demands at age 26 (similar procedure for decision latitude). Also, four cumulative exposure categories reflecting change in exposure to work demands and decision latitude were created at ages 22 and 26 to discover their potential association with mental health problems: stable high, stable low, high to low and low to high.

The selection of control variables was based on previous literature and included sex, age, educational attainment, marital status, job change, physical health and early life mental health problems. Sex was assessed at age 11. Participants’ age was their age at each measurement wave. Educational attainment was measured at ages 22 and 26 and categorized into low (primary, lower vocational and lower secondary education), medium (intermediate vocational and intermediate secondary education) and high (higher secondary, higher vocational education and university). Marital status was classified at ages 22 and 26 as married or cohabitating; long-standing relationship; or single. Job change was retrospectively measured between ages 22 and 26 (yes/no). Physical health was assessed at ages 22 and 26 on a five-point Likert-scale ranging from 1 = bad to 5 = excellent. Early life mental health problems were measured at ages 11, 13 and 16 with the Youth Self-Report (YSR) and at ages 19 and 22 with the ASR.^23^^,^^33^ The scoring follows the procedures as described above.

### Statistical analyses

First, participants were described based on their socio-demographic characteristics, PWCs and mental health problems. Second, linear regression analyses were conducted to examine the associations between single and cumulative exposure to PWCs (for work demands and decision latitude separately and for the demand/control quadrant) at ages 22 and 26 and mental health problems at age 29. For all regression analyses, we calculated two models: crude (Model 1), adjusted for sex, age, physical health, educational attainment, marital status, job change and for internalizing problems in the analyses on internalizing problems and for externalizing problems in the analyses on externalizing problems (Model 2). Three sensitivity analyses were conducted. In the first sensitivity analysis, we examined change in exposure to PWCs to discover their potential association with mental health problems. The second sensitivity analysis included only participants working more than 12 per week and not studying at ages 22 and 26 to examine the potential impact of being a student with a side job. The third sensitivity analysis included all working participants working any hours at ages 22 and 26 to examine potential selection bias. All statistical analyses were performed in SPSS version 26.

## Results

### Sample characteristics

Most young adults were female (65.5%), with a medium level of education at ages 22 and 26, single at age 22 and married or cohabitating at age 26, reported moderate to good physical health and changed jobs between ages 22 and 26 (66.0%) (table 1). At age 29, the mean scores for internalizing and externalizing problems were respectively 12.5 (SD 12.0) and 8.0 (SD 8.0). At ages 22 and 26, most young adults were working in an active job (36.5% and 32.6%, respectively) (table 2).

**Table 1:** Sample description of socio-demographic factors for the total sample and by internalizing and externalizing problems at age 29 (*N* = 362)

	Age^a^	Total sample, *N* (%)	Internalizing problem scores age 29, mean (SD)^b^	Externalizing problem scores age 29, mean (SD)^b^
Sex, *N* (%)	11			
Male		125 (34.5)	9.91 (10.5)	7.63 (8.4)
Female		237 (65.5)	13.88 (12.5)	7.56 (7.8)
Educational attainment, *N* (%)	22			
Low		27 (7.5)	16.52 (15.1)	11.06 (9.5)
Medium		277 (76.5)	12.49 (12.0)	7.68 (8.1)
High		58 (16.0)	10.70 (9.8)	5.49 (5.8)
Educational attainment, *N* (%)	26			
Low		18 (5.0)	15.74 (16.6)	13.33 (12.6)
Medium		183 (50.6)	13.57 (12.3)	8.45 (8.3)
High		161 (44.5)	10.93 (10.8)	5.96 (6.4)
Marital status	22			
Married or cohabitating		89 (24.6)	11.34 (11.4)	7.58 (8.7)
Long-standing relationship		115 (31.8)	11.51 (11.4)	6.47 (7.0)
Single		158 (43.6)	13.89 (12.7)	8.40 (8.2)
Marital status	26			
Married or cohabitating		202 (55.8)	10.64 (10.5)	6.83 (7.5)
Long-standing relationship		39 (10.8)	11.77 (9.6)	6.88 (5.6)
Single		121 (33.4)	15.85 (14.2)	9.07 (9.2)
Job change, *N* (%)	26			
Yes		239 (66.0)	13.09 (11.6)	7.87 (8.0)
No		123 (34.0)	11.36 (12.7)	7.03 (8.0)
Physical health, mean (SD)^c^	22	3.22 (0.8)		
Physical health, mean (SD)^c^	26	3.20 (0.8)		
Internalizing problems (mean, SD)^b^				
	11	17.84 (11.9)		
	13	16.71 (12.0)		
	16	16.63 (12.2)		
	19	10.90 (10.5)		
	22	11.53 (10.7)		
	29	12.50 (12.0)		
Externalizing problems (mean, SD)^b^				
	11	12.85 (9.8)		
	13	13.97 (9.0)		
	16	15.02 (9.7)		
	19	10.44 (8.8)		
	22	9.04 (7.9)		
	29	7.98 (8.0)		

a Age at which variable was measured.

b Range 0.00–100.

c Range 1.00–5.00.

**Table 2:** Sample description of psychosocial work conditions for the total sample and by internalizing and externalizing problems at age 29 (*N* = 362)

	Age^a^	Total sample, *N* (%)	Internalizing problem scores age 29, mean (SD)^b^	Externalizing problem scores age 29, mean (SD)^b^
**Single exposure**				
Work demands	22			
High		144 (39.8)	15.52 (14.1)	8.92 (7.9)
Medium		76 (21.0)	10.71 (8.3)	7.05 (7.8)
Low		142 (39.2)	10.41 (10.7)	6.52 (8.03)
High	26	138 (38.1)	14.82 (13.5)	8.12 (8.2)
Medium		120 (33.1)	10.67 (11.2)	6.75 (7.75)
Low		104 (28.7)	11.55 (10.2)	7.84 (7.9)
Decision latitude	22			
Low		116 (32.0)	14.93 (13.0)	8.15 (7.7)
Medium		106 (29.3)	11.16 (9.9)	7.41 (7.6)
High		140 (38.7)	11.51 (12.3)	7.24 (8.4)
Low	26	100 (27.6)	14.78 (12.0)	8.19 (7.9)
Medium		115 (31.8)	12.90 (12.0)	7.93 (8.7)
High		147 (40.6)	10.65 (10.8)	6.91 (7.4)
Demand/control model	22			
Low-strain		75 (20.7)	8.62 (9.4)	6.29 (8.9)
High-strain		88 (24.3)	15.21 (12.9)	8.60 (7.6)
Passive		67 (18.5)	12.42 (11.8)	6.78 (6.9)
Active		132 (36.5)	12.96 (12.4)	8.05 (8.1)
Low-strain	26	95 (26.2)	10.78 (10.1)	6.81 (6.8)
High-strain		69 (19.1)	14.81 (12.4)	8.57 (8.7)
Passive		80 (22.1)	11.83 (11.5)	7.64 (8.3)
Active		118 (32.6)	13.01 (13.3)	7.59 (8.2)
**Cumulative exposure**				
Work demands	22–26			
High		78 (21.5)	15.22 (14.9)	7.8 (7.0)
Medium		34 (9.4)	10.14 (8.7)	7.4 (8.6)
Other		250 (69.1)	11.98 (11.3)	7.5 (8.2)
Decision latitude	22–26			
Low		45 (12.4)	16.72 (14.1)	8.48 (8.4)
Medium		36 (9.9)	11.61 (10.4)	7.38 (7.7)
Other		281 (77.6)	11.94 (11.7)	7.47 (8.0)
Demand/control model	22–26			
High-strain		23 (6.4)	16.67 (12.7)	8.01 (7.0)
Active		67 (18.5)	13.02 (14.4)	8.29 (9.0)
Other		272 (75.1)	12.03 (12.0)	7.37 (7.8)
**Change of exposure**				
Work demands	22–26			
Stable high		139 (38.4)	13.80 (13.1)	8.07 (7.9)
High to low		81 (22.4)	13.96 (11.8)	8.62 (8.0)
Low to high		48 (13.3)	13.30 (12.7)	7.62 (9.9)
Stable low		94 (26.0)	8.93 (9.3)	5.96 (6.9)
Decision latitude	22–26			
Stable low		78 (21.5)	14.46 (12.8)	7.58 (7.6)
Low to high		77 (21.3)	13.54 (12.1)	8.05 (7.2)
High to low		71 (19.6)	11.83 (10.9)	8.61 (9.4)
Stable high		136 (37.6)	11.15 (11.9)	6.79 (7.8)

a Age at which variable was measured.

b Range 0.00–100.

### Single exposure to psychosocial work conditions

Table 3 shows that young adults with a single exposure in time to high work demands at ages 22 or 26 experience more internalizing problems than their peers in jobs with low work demands. After adjustment for sociodemographic variables and early life internalizing problems, the results attenuated, but remained significant (Model 2). Young adults in jobs with a single exposure to low decision latitude at ages 22 or 26 experience more internalizing problems than their peers with a single exposure in time to high decision latitude; after adjustment for sociodemographic variables and early life internalizing problems these results attenuated and became non-significant. Concerning the demand control model, young adults with a single exposure to high-strain or active jobs at age 22 experience more internalizing problems than their peers with a single exposure to low-strain jobs. After adjustment for sociodemographic variables and early life internalizing problems, the result for active jobs became non-significant, while the result for high-strain jobs attenuated but remained significant. Overall, the analyses of externalizing problems showed that none of the PWC measures were associated with externalizing problems after adjustment for early life externalizing problems.

**Table 3:** Regression analyses of single exposure to psychosocial work conditions at ages 22 and 26 on internalizing and externalizing problems at age 29 (*N* = 362)

			Internalizing problems		Externalizing problems	
	Age	Level	Model 1	Model 2	Model 1	Model 2
			β^a^ (95% CI^b^)	β^a^ (95% CI^b^)	β^a^ (95% CI^b^)	β^a^ (95% CI^b^)
Work demands	22	High	5.11 (2.37 to 7.85)	3.05 (0.63 to 5.46)	2.40 (0.56 to 4.24)	1.16 (−0.44 to 2.75)
		Medium	0.30 (−2.99 to 3.59)	−0.45 (−3.30 to 2.41)	0.53 (−1.68 to 2.74)	−0.40 (−1.93 to 1.85)
		Low	Ref.	Ref.	Ref.	Ref.
	26	High	3.27 (0.23 to 6.30)	2.78 (0.21 to 5.36)	0.27 (−1.76 to 2.31)	0.50 (−1.16 to 2.15)
		Medium	−0.88 (−4.01 to 2.25)	0.24 (−2.42 to 2.89)	−1.09 (−3.20 to 1.01)	−0.51 (−2.22 to 1.19)
		Low	Ref.	Ref.	Ref.	Ref.
Decision latitude	22	Low	3.42 (0.48 to 6.36)	1.01 (−1.58 to 3.60)	0.91 (−1.06 to 2.88)	0.32 (−1.38 to 2.01)
		Medium	−0.35 (−3.36 to 2.67)	−1.29 (−3.90 to 1.32)	0.17 (−1.86 to 2.19)	−0.38 (−2.10 to 1.34)
		High	Ref.	Ref.	Ref.	Ref.
	26	low	4.13 (1.10 to 7.17)	2.59 (−0.03 to 5.21)	1.28 (−0.76 to 3.31)	1.45 (−0.23 to 3.12)
		Medium	2.25 (−0.66 to 5.16)	1.59 (−1.84 to 4.03)	1.02 (−0.94 to 2.97)	0.60 (−0.98 to 2.17)
		High	Ref.	Ref.	Ref.	Ref.
Demand/control model	22	High-strain	6.59 (2.94 to 10.25)	3.32 (0.09 to 6.56)	2.32 (−0.14 to 4.78)	0.86 (−1.26 to 2.98)
		Passive	3.80 (−0.11 to 7.71)	2.24 (−1.17 to 5.65)	0.50 (−2.14 to 3.13)	0.23 (−2.04 to 2.49)
		Active	4.34 (0.98 to 7.70)	2.61 (−0.37 to 5.60)	1.77 (−0.50 to 4.03)	0.83 (−1.14 to 2.81)
		Low-strain	Ref.	Ref.	Ref.	Ref.
	26	High-strain	4.03 (0.31 to 7.75)	2.57 (−0.62 to 5.76)	1.76 (−0.73 to 4.24)	2.06 (0.03 to 4.09)
		Passive	1.04 (−2.52 to 4.61)	1.16 (−1.83 to 4.16)	0.83 (−1.55 to 3.21)	1.38 (−0.55 to 3.31)
		Active	2.22 (−1.02 to 5.46)	1.90 (−0.83 to 4.62)	0.78 (−1.39 to 2.94)	0.83 (−0.90 to 2.58)
		Low-strain	Ref.	Ref.	Ref.	Ref.

Model 1: crude | Model 2: adjusted for sex, age, physical health, educational attainment, marital status, job change, childhood and adolescence mental health problems (internalizing problems for the analyses on internalizing problems and externalizing problems for the analyses on externalizing problems).

a Unstandardized beta coefficient.

b 95% confidence interval.

### Cumulative exposure to psychosocial work conditions

Table 4 shows that cumulative exposure to PWCs at both ages 22 and 26 was not associated with internalizing or externalizing problems at age 29 after adjusting for sociodemographic variables and early life internalizing or externalizing problems.

**Table 4:** Regression analyses of cumulative exposure to psychosocial work conditions at ages 22 and 26 on internalizing and externalizing problems at age 29 (*N* = 362)

		Internalizing problems		Externalizing problems	
	Level	Model 1	Model 2	Model 1	Model 2
		β^a^ (95% CI^b^)	β^a^ (95% CI^b^)	β^a^ (95% CI^b^)	β^a^ (95% CI^b^)
Work demands	High	3.24 (0.20 to 6.28)	2.11 (−0.44 to 4.66)	0.31 (−1.73 to 2.35)	0.11 (−1.54 to 1.77)
	Medium	−1.84 (−6.12 to 2.45)	−1.65 (−5.22 to 1.93)	−0.09 (−2.96 to 2.79)	0.63 (−1.68 to 2.94)
	Other	Ref.	Ref.	Ref.	Ref.
Decision latitude	Low	4.78 (1.02 to 8.54)	1.70 (−1.54 to 4.94)	1.01 (−1.52 to 3.53)	0.80 (−1.26 to 2.86)
	Medium	−0.34 (−4.48 to 3.81)	−0.99 (−4.51 to 2.53)	−0.09 (−2.87 to 2.69)	−0.60 (−2.85 to 1.65)
	Other	Ref.	Ref.	Ref.	Ref.
Demand/control model	High-strain	4.64 (−0.47 to 9.75)	1.82 (−2.58 to 6.23)	0.64 (−2.77 to 4.05)	−0.62 (−3.41 to 2.17)
	Active	0.98 (−2.23 to 4.19)	0.90 (−1.83 to 3.62)	0.92 (−1.22 to 3.06)	−0.21 (−1.97 to 1.55)
	Other	Ref.	Ref.	Ref.	Ref.

Model 1: crude | Model 2: adjusted for sex, age, physical health, educational attainment, marital status, job change, childhood and adolescence mental health problems (internalizing problems for the analyses on internalizing problems and externalizing problems for the analyses on externalizing problems).

a Unstandardized beta coefficient.

b 95% confidence interval.

### Sensitivity analyses

Changes in exposure to work demands at ages 22 and 26 were associated with internalizing problems at age 29, but attenuated and became non-significant after adjustment for early life internalizing problems (table 3). The analysis, in which the sample of only participants working more than 12 hours per week and not studying at ages 22 and 26 (*N* = 167) was compared with the final sample (*N* = 362), revealed minor differences with the results of the main analysis (Supplementary tables S1 and S2). Regarding single exposure to PWCs, exposure to high work demands at age 26 was not associated with internalizing problems at age 29. Regarding cumulative exposure to PWCs, exposure to high-strain jobs at both ages 22 and 26 was associated with internalizing problems, but these results attenuated and became non-significant after adjustment for sociodemographic variables and early life internalizing problems. The sensitivity analysis with all working participants at ages 22 and 26 (*N* = 608) revealed minor differences with the results of the main analysis (*N* = 362) (Supplementary tables S3 and S4).

## Discussion

Our prospective study showed that a single exposure at one point in time to high work demands at either ages 22 or 26 and high-strain jobs at age 22 was associated with internalizing problems among young adults at age 29. The associations persisted after adjustment for sociodemographic and work covariates. As hypothesized, the associations attenuated after adjustment for early life internalizing and externalizing problems. Cumulative exposure to PWCs was not associated with internalizing or externalizing problems. To our knowledge, this is the first comprehensive study of the prospective association between single and cumulative exposures to PWCs and subsequent mental health problems among young adults that controlled for early life mental health problems.

In line with existing studies, we found evidence for the impact of single exposure in time to PWCs for internalizing problems.^13^^,^^14^^,^^16^ However, the associations between single exposure to PWCs and internalizing problems in our study should be considered rather weak.^34^ The finding of weak associations in a relatively healthy sample of young workers, indicates that the single exposure to PWCs might not be clinically relevant. More research on PWCs among larger populations of young adults is needed to further investigate the associations and underlying mechanisms between PWCs and mental health in greater detail.

In contrast to the few studies that have examined the cumulative exposure of PWCs, we did not find associations between cumulative exposures and mental health problems among young adults, perhaps due to a relatively small sample size. However, the sensitivity analysis with a larger sample (*N* = 608) also showed no impact of cumulative exposure to PWCs on subsequent mental health problems. Our findings indicate that the impact of PWCs is weaker at age 26 than at age 22. An explanation might be that young workers have left their job or have adapted to their PWCs, for example by learning how to cope with their adverse PWCs.^35^ Another explanation might be that previous studies focused on specific groups, such as older workers or student workers in high school or vocational training,^13^^,^^14^^,^^16^ which may explain the different findings. Also, the studies focusing on student workers differ in the operationalization of mental health problems (i.e. depressive affect and rumination) and PWCs (i.e. stress-oriented tasks and work stressor index scores).^14^^,^^16^ Our operationalization of mental problems and PWCs is most comparable to the study among the older workforce.^13^ While Stansfeld’s and our study used Karasek’s job demands-control model,^31^ it is questionable whether results from two decades ago are replicable to young workers anno 2020. Today’s young workers face more temporary work, multiple jobs, self-employment and a 24/7 work cycle in a global economy.^36^ It could be that as the world of work has changed, so have the perceptions of today’s young workers. Although Karasek’s (1979) job demands-control model is one of the most widely studied models of occupational stress, it may no longer capture today’s young adults’ psychosocial work environment. Future research addressing Karasek’s job demands-control model among young workers is needed to explore this in more detail.

A strength of our study is the prospective design with rigorous repeated, temporally ordered measures that allowed us to examine the associations between single and cumulative exposure to adverse PWCs for today’s young workers’ mental health. We measured PWCs at two measurement points, which provided more detailed exposure information on our transitioning young general working population. Another strength concerns the use of the life course approach by controlling for early life mental health problems from childhood to adolescence. Other studies may have overestimated the association between PWCs and mental health by not controlling for early life mental health problems.

When interpreting the results, the following methodological issues must be considered. We excluded a large proportion of participants who worked <12 h per week at ages 22 and 26. The sensitivity analysis, including all working participants at ages 22 and 26, showed minor differences with our main analyses, indicating that selection bias is unlikely. Also, the current study measured PWCs at two time points over a 5-year time span, but in an ideal world, we would like to measure exposure more frequently during follow-up. For a better understanding of the impact of PWCs on subsequent mental health problems among young adults, more research is needed on the exposure to PWCs experience. Another limitation is that we were not able to stratify by sex due to sample size issues. Previous research has shown that men tend to report more externalizing problems, whereas women report more internalizing problems.^37–39^ Future research with larger samples should perform sex-stratified analyses to examine possible sex differences in the associations between PWCs and mental health in young adults.

Our findings have implications for research, policy and practice. Future research on PWCs and mental health problems among young adults may apply a life course approach, including repeated measures of PWCs over a longer period of time, to examine whether the effects of accumulation of adverse exposure to PWCs on mental health change over time. As work demands represent potentially modifiable work conditions, we recommend that programmes should be developed to decrease work demands to prevent or at least reduce mental health problems and more severe consequences, such as sickness absence and work disability among young workers. Valid instruments to measure PWCs, such as the Copenhagen Psychosocial Questionnaire (COPSOQ)^25^ or the newly developed Danish Psychosocial Work Environment Questionnaire (DPQ)^40^ may serve as starting points for occupational physicians to identify potential adverse PWCs that can be accommodated to promote the mental health of young workers. Furthermore, the life course approach in our study illustrates the importance of taking early life mental health problems into consideration. Therefore, policymakers and practitioners in the youth and occupational health domains should pay attention to pre-work mental health experiences, as young workers' mental health does not start when they start working.

In conclusion, single exposures in time, not cumulative exposure, to high work demands and high-strain jobs were associated with internalizing problems among young adults. Our findings illustrate the importance of using a life course approach when investigating the association between PWCs and subsequent mental health problems.

## Supplementary Material

ckad015_Supplementary_Data

## Data Availability

Data may be obtained from a third party and are not publicly available. TRAILS data of the T1, T2, T3, T4 and T5 measurement waves are deposited in the Data Archiving and Networked Services of the Royal Dutch Academy of Sciences (DANS-KNAW) and access can be requested at “http://www.dans.knaw.nl”.
