# Supplementary material for: Single and cumulative exposure to psychosocial work conditions and mental health among young adults
Source: Eur J Public Health. 2023 Feb 15;33(2):257–63. doi: 10.1093/eurpub/ckad015 (PMC10066491; doi:10.1093/eurpub/ckad015)
Supplement: ckad015_Supplementary_Data [file ckad015_supplementary_data.docx]

**Supplemental material**

*Table S1: Regression analyses of change of exposure to psychosocial work conditions at ages 22 and 26 on internalising and externalising problems at age 29 (N=362)*

|  |  | Internalising problems | | Externalising problems | |
| --- | --- | --- | --- | --- | --- |
|  | Level | Model 1 | Model 2 | Model 1 | Model 2 |
|  |  | β^a^ (95% CI^b^) | β^a^ (95% CI^b^) | β^a^ (95% CI^b^) | β^a^ (95% CI^b^) |
| Work demands | Stable high | 4.87 (1.75-7.98) | 2.30 (-0.38-4.98) | 2.11 (0.02-4.20) | 0.64 (-1.10-2.37) |
|  | High to low | 5.03 (1.49-8.56) | 1.93 (-1.18-5.04) | 2.67 (0.30-5.04) | 0.68 (-1.29-2.64) |
|  | Low to high | 4.37 (0.24-8.50) | 2.49 (-1.12-6.09) | 1.66 (-1.11-4.43) | 1.40 (-0.92-3.72) |
|  | Stable low | Ref. | Ref. | Ref. | Ref. |
| Decision latitude | Stable low | 3.31 (-0.03-6.65) | 1.52 (-1.35-4.40) | 0.80 (-1.43-3.03) | 1.21 (-0.61-3.02) |
|  | Low to high | 2.39 (-0.97-5.74) | 0.73 (-2.12-3.58) | 1.27 (-0.97-3.50) | 1.04 (-0.77-2.84) |
|  | High to low | 0.68 (-2.77-4.12) | 0.10 (-2.91-3.11) | 1.83 (-0.47-4.12) | 2.37 (0.45-4.30) |
|  | Stable high | Ref. | Ref. | Ref. | Ref. |

*Model 1: crude | Model 2: adjusted for sex, age, physical health, educational attainment, marital status, job change, childhood and adolescence mental health problems (internalising problems for the analyses on internalising problems and externalising problems for the analyses on externalising problems)*

*a: unstandardized beta coefficient*

*b: 95% confidence interval*

*Table S2: Regression analyses of single exposure to psychosocial work conditions for participants working more than 12 hours per week and not studying at ages 22 and 26 on internalising and externalising problems at age 29 (N=167)*

|  |  |  | Internalising problems | | Externalising problems | |
| --- | --- | --- | --- | --- | --- | --- |
|  | Age | Level | Model 1 | Model 2 | Model 1 | Model 2 |
|  |  |  | β^a^ (95% CI^b^) | β^a^ (95% CI^b^) | β^a^ (95% CI^b^) | β^a^ (95% CI^b^) |
| Work demands | 22 | High | 4.71 (0.78-8.64) | 2.05 (-1.55-5.65) | 1.12 (-1.70-3.93) | -0.13 (-2.75-2.49) |
|  |  | Medium | 2.75 (-1.68-7.18) | 2.00 (-1.93-5.93) | 1.40 (-1.77-4.57) | 1.21 (-1.63-4.06) |
|  |  | Low | Ref. | Ref. | Ref. | Ref. |
|  | 26 | High | 3.32 (-0.89-7.52) | 1.93 (-1.76-5.62) | 0.68 (-2.31-3.67) | 0.04 (-2.61-2.68) |
|  |  | Medium | 0.31 (-3.99-4.61) | 0.43 (-3.29-4.15) | -0.53 (-3.56-2.55) | -0.33 (-3.01-2.34) |
|  |  | Low | Ref. | Ref. | Ref. | Ref. |
| Decision latitude | 22 | Low | 7.75 (3.56-11.95) | 5.60 (1.72-9.48) | 2.33 (-0.75-5.41) | 2.10 (-0.76-4.96) |
|  |  | Medium | -0.50 (-4.36-3.37) | -0.32 (-3.83-3.20) | -0.52 (-3.35-2.32) | -0.63 (-3.23-1.97) |
|  |  | High | Ref. | Ref. | Ref. | Ref. |
|  | 26 | low | 3.71 (-0.51-7.94) | 3.71 (0.07-7.35) | 1.80 (-1.17-4.77) | 2.58 (-0.04-5.19) |
|  |  | Medium | 3.52 (-0.59-7.63) | 1.86 (-1.60-5.32) | 3.36 (0.47-6.25) | 1.74 (-0.85-4.32) |
|  |  | High | Ref. | Ref. | Ref. | Ref. |
| Demand/control model | 22 | High-strain | 9.31 (4.33-14.30) | 6.74 (2.28-11.20) | 2.75 (-0.88-6.39) | 1.83 (-1.50-5.16) |
|  |  | Passive | 3.62 (-1.62-8.86) | 3.06 (-1.51-7.63) | 1.14 (-2.68-4.97) | 1.23 (-2.24-4.70) |
|  |  | Active | 3.21 (-1.21-7.62) | 1.30 (-2.65-5.25) | 1.11 (-2.11-4.33) | 0.47 (-2.50-3.44) |
|  |  | Low-strain | Ref. | Ref. | Ref. | Ref. |
|  | 26 | High-strain | 3.48 (-1.58-8.54) | 2.59 (-1.77-6.95) | 2.72 (-0.85-6.28) | 3.18 (0.09-6.27) |
|  |  | Passive | 1.72 (-3.10-6.54) | 1.70 (-2.41-5.81) | 0.83 (-2.57-4.22) | 1.51 (-1.43-4.46) |
|  |  | Active | 2.25 (-2.37-6.86) | 0.61 (-3.35-4.57) | 0.39 (-2.86-3.64) | -0.01 (-2.83-2.80) |
|  |  | Low-strain | Ref. | Ref. | Ref. | Ref. |

*Model 1: crude | Model 2: adjusted for sex, age, physical health, educational attainment, marital status, job change, childhood and adolescence mental health problems problems (internalising problems for the analyses on internalising problems and externalising problems for the analyses on externalising problems)*

*a: unstandardized beta coefficient*

*b: 95% confidence interval*

*Table S3: Regression analyses of cumulative exposure to psychosocial work conditions for participants working more than 12 hours per week and not studying at ages 22 and 26 on internalising and externalising problems at age 29 (N=167)*

|  |  |  | Internalising problems | |  | Externalising problems | |
| --- | --- | --- | --- | --- | --- | --- | --- |
|  | Level | Model 1 | | Model 2 | | Model 1 | Model 2 |
|  |  | β^a^ (95% CI^b^) | | β^a^ (95% CI^b^) | | β^a^ (95% CI^b^) | β^a^ (95% CI^b^) |
| Work demands | High | 1.67 (-2.73-6.06) | | -0.51 (-4.28-3.26) | | -0.77 (-3.86-2.32) | -2.08 (-4.78-0.61) |
|  | Medium | 1.22 (-4.49-6.92) | | 1.74 (-3.15-6.63) | | 2.14 (-1.87-6.15) | 2.23 (-1.24-5.70) |
|  | Other | Ref. | | Ref. | | Ref. | Ref. |
| Decision latitude | Low | 6.28 (0.83-11.74) | | 4.28 (-0.70-9.26) | | 1.84 (-2.05-5.74) | 3.16 (-0.40-6.71) |
|  | Medium | -0.59 (-5.81-4.63) | | -0.39 (-4.94 -4.17) | | 0.68 (-3.05-4.42) | 0.05 (-3.23-3.34) |
|  | Other | Ref. | | Ref. | | Ref. | Ref. |
| Demand/control model | High-strain | 10.82 (2.79-18.85) | | 6.51 (-0.57-13.60) | | 2.08 (-3.70-7.85) | -0.38 (-5.68-4.92) |
|  | Active | -0.76 (-5.29-3.76) | | -2.67 (-6.65-1.31) | | -0.41 (-3.66-2.85) | -2.03 (-4.93-0.87) |
|  | Other | Ref. | | Ref. | | Ref. | Ref. |

*Model 1: crude | Model 2: adjusted for sex, age, physical health, educational attainment, marital status, job change, childhood and adolescence mental health problems (internalising problems for the analyses on internalising problems and externalising problems for the analyses on externalising problems)*

*a: unstandardized beta coefficient*

*b: 95% confidence interval*

*Table S4: Regression analyses of single exposure to psychosocial work conditions for participants working any hours at ages 22 and 26 on internalising and externalising problems at age 29 (N=608)*

|  |  |  | Internalising problems |  | Externalising problems | |
| --- | --- | --- | --- | --- | --- | --- |
|  | Age | Level | Model 1 | Model 2 | Model 1 | Model 2 |
|  |  |  | β^a^ (95% CI^b^) | β^a^ (95% CI^b^) | β^a^ (95% CI^b^) | β^a^ (95% CI^b^) |
| Work demands | 22 | High | 4.46 (2.18-6.73) | 1.95 (-0.01-3.90) | 3.14 (1.64-4.64) | 1.31 (0.04-2.58) |
|  |  | Medium | 0.46 (-2.25-3.18) | -1.11 (-3.42-1.20) | 1.09 (-0.70-2.88) | -0.17 (-1.67-1.34) |
|  |  | Low | Ref. | Ref. | Ref. | Ref. |
|  | 26 | High | 3.21 (0.69-5.73) | 2.11 (0.04-4.18) | 0.25 (-1.43-1.92) | 0.23 (-1.12-1.57) |
|  |  | Medium | -0.55 (-3.15-2.05) | -0.05 (-2.17-2.08) | -0.96 (-2.69-0.77) | -0.39 (-1.78-1.00) |
|  |  | Low | Ref. | Ref. | 0.25 (-1.43-1.92) | Ref. |
| Decision latitude | 22 | Low | 3.52 (1.14-5.90) | 2.31 (0.30-4.32) | 1.20 (-0.38-2.78) | 0.86 (-0.45-2.17) |
|  |  | Medium | 1.22 (-1.49-3.93) | 0.23 (-2.06-2.52) | 1.03 (-0.77-2.82) | 0.46 (-1.03-1.95) |
|  |  | High | Ref. | Ref. | Ref. | Ref. |
|  | 26 | low | 5.23 (2.76-7.70) | 2.89 (0.81-4.97) | 1.88 (0.23-3.52) | 1.05 (-0.30-2.40) |
|  |  | Medium | 1.67 (-0.75-4.09) | 0.76 (-1.21-2.72) | 0.53 (-1.09-2.15) | -0.02 (-1.30-1.27) |
|  |  | High | Ref. | Ref. | Ref. | Ref. |
| Demand/control model | 22 | High-strain | 6.14 (3.17-9.11) | 2.90 (0.35-5.44) | 3.00 (1.03-4.96) | 1.13 (-0.52-2.79) |
|  |  | Passive | 3.55 (0.42-6.67) | 2.10 (-0.54-4.73) | -0.11 (-2.17-1.96) | -0.18 (-1.89-1.54) |
|  |  | Active | 3.73 (0.70-6.76) | 1.04 (-1.55-3.63) | 1.63 (-0.37-3.63) | 0.16 (-1.52-1.85) |
|  |  | Low-strain | Ref. | Ref. | Ref. | Ref. |
|  | 26 | High-strain | 5.06 (2.03-8.09) | 2.61 (0.11-5.11) | 1.67 (-0.35-3.68) | 0.98 (-0.64-2.60) |
|  |  | Passive | 2.51 (-0.45-5.47) | 1.53 (-0.89-3.94) | 1.00 (-0.97-2.97) | 0.71 (-0.87-2.28) |
|  |  | Active | 1.72 (-0.98-4.43) | 0.96 (-1.24-3.16) | 0.24 (-1.56-2.03) | 0.29 (-1.14-1.72) |
|  |  | Low-strain | Ref. | Ref. | Ref. | Ref. |

*Model 1: crude | Model 2: adjusted for sex, age, physical health, educational attainment, marital status, job change, childhood and adolescence mental health problems (internalising problems for the analyses on internalising problems and externalising problems for the analyses on externalising problems)*

*a: unstandardized beta coefficient*

*b: 95% confidence interval*

*Table S5: Regression analyses of cumulative exposure to psychosocial work conditions for participants working any hours at ages 22 and 26 on internalising and externalising problems at age 29 (N=608)*

|  |  | Internalising problems | |  | Externalising problems | |
| --- | --- | --- | --- | --- | --- | --- |
|  | Level | Model 1 | Model 2 | | Model 1 | Model 2 |
|  |  | β^a^ (95% CI^b^) | β^a^ (95% CI^b^) | | β^a^ (95% CI^b^) | β^a^ (95% CI^b^) |
| Work demands | High | 3.08 (0.47-5.68) | 1.81 (-0.29-3.91) | | 0.98 (-0.75-2.71) | 0.61 (-0.76-1.98) |
|  | Medium | -2.68 (-6.39-1.04) | -2.04 (-5.03-0.94) | | -1.07 (-3.54-1.40) | 0.01 (-1.95-1.96) |
|  | Other | Ref. | Ref. | | Ref. | Ref. |
| Decision latitude | Low | 6.02 (3.28-8.76) | 2.37 (0.07-4.68) | | 2.19 (0.37-4.02) | 0.69 (-0.80-2.19) |
|  | Medium | 0.71 (-2.87-4.30) | -0.23 (-3.15-2.69) | | 0.53 (-1.87-2.92) | -0.25 (-2.16-1.66) |
|  | Other | Ref. | Ref. | | Ref. | Ref. |
| Demand/control model | High-strain | 5.48 (1.88-9.08) | 1.66 (-1.29-4.62) | | 2.64 (0.25-5.03) | 0.30 (-1.62-2.12) |
|  | Active | 0.03 (-3.01-3.06) | -0.41 (-2.90-2.09) | | 0.72 (-1.29-2.73) | -0.37 (-2.00-1.26) |
|  | Other | Ref. | Ref. | | Ref. | Ref. |

*Model 1: crude | Model 2: adjusted for sex, age, physical health, educational attainment, marital status, job change, childhood and adolescence mental health problems (internalising problems for the analyses on internalising problems and externalising problems for the analyses on externalising problems)*

*a: unstandardized beta coefficient*

*b: 95% confidence interval*
